# Nonlinear Optical Phenomena in a Silicon-Smectic A Liquid Crystal (SALC) Waveguide

**DOI:** 10.3390/ma12132086

**Published:** 2019-06-28

**Authors:** Boris I. Lembrikov, David Ianetz, Yosef Ben-Ezra

**Affiliations:** Faculty of Electrical Engineering, Holon Institute of Technology, P.O. Box 305, 52 Golomb str., Holon 58102, Israel

**Keywords:** silicon photonics, optical waveguide, smectic A liquid crystal (SALC), stimulated light scattering (SLS)

## Abstract

Liquid crystals (LCs) are organic materials characterized by the intermediate properties between those of an isotropic liquid and a crystal with a long range order. The LCs possess strong anisotropy of their optical and electro-optical properties. In particular, LCs possess strong optical nonlinearity. LCs are compatible with silicon-based technologies. Due to these unique properties, LCs are promising candidates for the development of novel integrated devices for telecommunications and sensing. Nematic liquid crystals (NLCs) are mostly used and studied. Smectic A liquid crystals (SALCs) have a higher degree of long range order forming a layered structure. As a result, they have lower scattering losses, specific mechanisms of optical nonlinearity related to the smectic layer displacement without the mass density change, and they can be used in nonlinear optical applications. We theoretically studied the nonlinear optical phenomena in a silicon-SALC waveguide. We have shown theoretically that the stimulated light scattering (SLS) and cross-phase modulation (XPM) caused by SALC nonlinearity can occur in the silicon-SALC waveguide. We evaluated the smectic layer displacement, the SALC hydrodynamic velocity, and the slowly varying amplitudes (SVAs) of the interfering optical waves.

## 1. Introduction

Liquid crystals (LCs) are promising candidates for applications in novel integrated devices for telecommunications, sensing, and lab-on-chip bioscience [1]. These applications are based on the unique optical properties of LC. The orientational energy of LC molecules is comparatively small, and for this reason they are characterized by an easy susceptibility to external field perturbation [2]. As a result, the LC effective refractive index can be controlled by an external electric field which may be used for optical transmission, reflection, switching, and modulation applications [2]. LCs are highly nonlinear optical materials because their properties such as temperature, molecular orientation, density, and electronic structure can be easily perturbed by an applied optical field [2].

The liquid crystal on silicon technology (LCOS) is widely used in telecommunications [3,4]. The basic element of the LCOS technology is the LCOS cell consisting of the LCOS backplane, LC layer and cover glass [3,4]. The LCOS cell can simultaneously perform the electrical and optical functions [3,4]. The photonic applications of the LCOS devices include the spatial light modulation (SLM), the holographic beam steering, optical wavelength selective switching, and the optical power control [4]. The LCOS SLM technology is a promising candidate for the so-called structured light where the optical field amplitude, phase, and polarization can be controlled spatially while the time and frequency spectrum can be controlled temporally [3]. Nonlinear silicon photonics can be used in on-chip optical signal processing and computation due to its low cost and compatibility with CMOS technology [5]. The development of nonlinear silicon photonics is limited by the absence of the second-order nonlinear susceptibility $\chi^{\left(2\right)}$ due to centrosymmetric structure of Si, comparatively low third-order nonlinear susceptibility $\chi^{\left(3\right)}$, the two-photon absorption (TPA) and free carrier absorption (FCA) [5]. To mitigate these disadvantages new materials with better nonlinear properties may be integrated with silicon. In such a case, the new materials may improve the nonlinearity of an optical device while silicon can confine the optical modes to nanoscale [5]. The organic nonlinear materials with a large $\chi^{\left(3\right)}$ can be used for the creation of a silicon-organic hybrid waveguide [5]. In particular, liquid crystals (LCs) may be used as a waveguide core where the modulation and switching of photonic signals is possible by using electro-optic or nonlinear optic effects [1,6,7,8,9,10,11,12,13].

We briefly discuss the basic properties of LCs. LCs are characterized by the properties intermediate between solid crystalline and liquid phases [2,14]. LCs flow like liquids, but possess a partial long range order and anisotropy of their physical parameters such as dielectric constants, elastic constants, viscosities, nonlinear susceptibilities [2]. Various phases in which such materials can exist are called mesophases [2]. There are three types of LCs: thermotropic LCs, polymeric LCs, and lyotropic LCs [2,14].

(1) Lyotropic LCs can be obtained in a solution with an appropriate concentration of a material.
(2) Polymeric LCs are the polymers consisting of the monomer LC molecules.
(3) Thermotropic LCs self-assemble in various ordered arrangement of their crystalline axis depending on the temperature.

Thermotropic LCs are most widely used and studied because of their extraordinary linear, electro-optical, and nonlinear optical properties and the possibility to control the transitions between different mesophases by varying the operating temperature [1,2]. The thermotropic LCs consist of elongated molecules with the direction of their axes determined by the unit vector $\vec{n}$ called director [2,14]. The long range ordering of LC mesophase is characterized by the director spatial distribution [2,14]. There exist three main types of thermotropic LCs: nematic LC (NLC), cholesteric LC (CLC), and smectic LC (SLC) [2,14]. NLC molecules are centrosymmetric in such a way that $\vec{n}$ and $-\vec{n}$ are equivalent, the molecules are positionally random, but they are mostly aligned in the direction defined by the director $\vec{n}$ [2,14]. CLC consists of chiral molecules, or they may be obtained by adding of chiral molecules to NLC [2]. As a result, they exhibit a helical structure where the direction $\vec{n}$ of the molecular orientation rotates in space around the helical axis with a period of about 300 nm [2]. The phase transition between the nematic and smectic A phases had been investigated both theoretically and experimentally in a large number of publications (see, for example, [2,15,16,17,18,19,20,21,22]). It is essentially the second kind phase transition [18]. The phase transition temperature $T_{SmA-N}$ may be different for different LC materials. For example, for 8CB $T_{SmA-N}\approx 307$ K, for 9CB $T_{SmA-N}\approx 321$ K [22].

SLC are characterized by the positional long range order in the direction of the elongated molecular axis and possess a layered structure with a layer thickness of about 2 nm approximately equal to the length of a SLC molecule [2,14]. Inside the layers, molecules are not ordered and represent a two-dimensional liquid [2,14]. There are different SLC phases [2,14].

(1) Smectic A LC (SALC) where the long axes of the molecules are perpendicular to the layer plane.
(2) Smectic B LC where the hexagonal in-layer ordering of the molecules perpendicular to the layer plane exists.
(3) Smectic C LC where the molecules are tilted with respect to the layers.
(4) Smectic C${}^{*}$ LC consisting of the chiral molecules and possessing the spontaneous polarization.
(5) So-called exotic smectic phases.

The nonlinear optical phenomena such as degenerate and nondegenerate wave mixing, optical bistabilities and instabilities, self-focusing and self-guiding, phase conjugation, stimulated light scattering (SLS), optical limiting, interface switching, beam combining, and self-starting laser oscillations have been observed in liquid crystalline materials [14,23]. NLCs are mainly studied and used in linear and nonlinear optical applications [2,4,23]. For instance, in NLC the optically induced director axis reorientation results in the so-called giant optical nonlinearity (GON) with the nonlinear refractive index coefficient $n_{2}^{NL}∼10^{-4}-10^{-3}$ cm${}^{2}$/W [14]. However, NLCs are characterized by large losses and relatively slow responses limiting their integrated electro-optical applications [2]. The light scattering properties of SALC thin film waveguide have been studied both theoretically and experimentally [24]. The scattering losses in smectic waveguides caused by dynamic distortions of the smectic layer planes are several orders of magnitude lower than in nematic waveguides [2,24], and SLCs may be used in nonlinear optical applications [2]. Recently, the reconfigurable smectic layer curvature has been studied [25]. The using of the external electric field to create the dynamic variations of the smectic layer configuration attracted a wide interest [25]. The different types of the periodic focal conic domain (FCD) arrays with the domain size, shape, orientation, and lattice symmetry controlled by external fields can be obtained [25].The applications of SALC such as soft-lithographic templates, superhydrophobic surfaces, microlens arrays, and optically selective photomasks have been developed [25].

The nonlinear optical phenomena in SALC have been investigated theoretically [26,27,28,29,30,31,32,33,34,35,36]. It has been shown that the light self-focusing, self-trapping, Brillouin-like SLS, and four-wave mixing (FWM) related to the light enhanced smectic layer normal displacement $u\left(\vec{r},t\right)$ occur in SALC under certain conditions. The nonlinear effects based on this nonlinearity mechanism specific for SALC are strongly anisotropic, and the corresponding SLS gain coefficient is significantly larger than the one in the case of the Brillouin SLS in isotropic organic liquids. The nonlinear interaction of the surface plasmon polaritons (SPPs) in the metal-insulator-metal (MIM) waveguide has been analyzed [36]. In particular, it has been shown theoretically that the strong SLS of the transverse magnetic (TM) even modes can occur in the optical slab waveguide with a SALC core [35].

In this paper, we investigated in detail SLS in the Silicon-SALC slab waveguide. We discussed in detail the peculiarities of different types of LCs and concentrated on the optical properties of SALC. We derived the SALC layer equation of motion and the truncated equations for the optical wave slowly varying amplitudes (SVAs). We discussed the contribution of the TM even and odd modes and the transverse electric (TE) modes of the Silicon-SALC waveguide. We solved simultaneously the Maxwell equations including the nonlinear polarization for the waveguide modes and the equation of motion for the smectic layer normal displacement $u\left(\vec{r},t\right)$ in the optical wave field. We evaluated $u\left(\vec{r},t\right)$ and the hydrodynamic velocity $\vec{v}\left(\vec{r},t\right)$ in the SALC core of the waveguide. We obtained the novel explicit solutions for the SVAs of the interfering waveguide modes and made numerical estimations of the waveguide mode parameters and the gain. The results of the numerical estimations are presented in Figures 2–8. The paper is constructed as follows. The theoretical model is presented in Section 2. The nonlinear polarization in the waveguide SALC core is evaluated in Section 3. The SVAs of the pumping and signal TM waveguide modes and the hydrodynamic velocity of smectic layers are calculated in Section 4. The conclusions are presented in Section 5.

## 2. Theoretical Model

**Figure 1:**
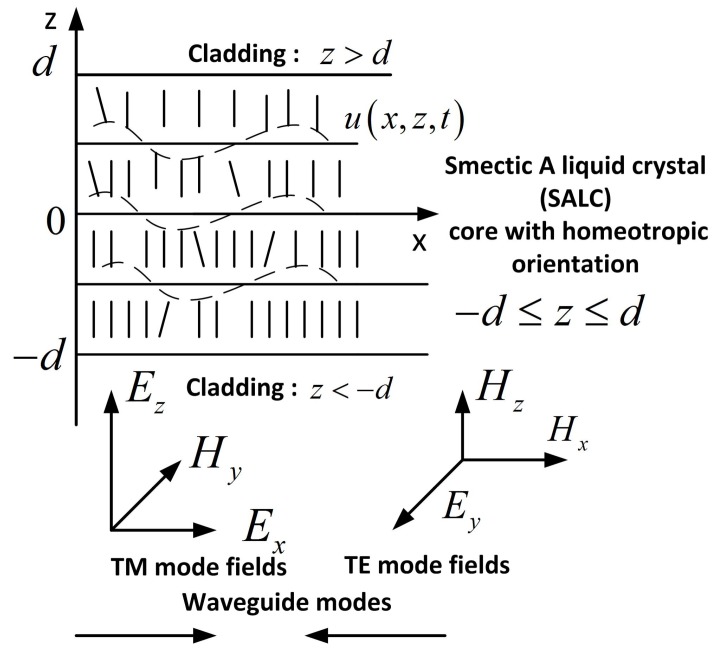
Optical slab waveguide with the homeotropically oriented SALC core of the thickness 2d. $E_{x,z},H_{y}$ and $E_{y},H_{x,z}$ are the electric and magnetic fields of the TM and TE modes, respectively.

A typical LC slab waveguide represents a LC thin film with a thickness of about 1 μm sandwiched between two glass slides of lower refractive index than LC [2]. One of slides is covered with an organic film. The input laser radiation is inserted into the film via the coupling prism [2]. The laser excites the TE and/or TM modes in the film which are then introduced into the LC core [2]. Such a structure can be placed on a Si substrate [8]. One of the claddings can be made of SiO${}_{2}$ [8]. For the sake of definiteness, we consider the homeotropically oriented SALC core where the molecular elongated axes are perpendicular to the waveguide claddings and the smectic layer planes parallel to them. The structure of the optical slab waveguide with a homeotropically oriented SALC core is shown in Figure 1.

Optical waves interact through the nonlinear polarization in a medium [37]. Generally, different types of SLS are described by the coupled wave equations for the light waves and for the corresponding material excitations [37]. The wave equation for electric field $\vec{E}\left(\vec{r},t\right)$ of the optical wave propagating in a nonlinear medium has the form [37].

(1) $$\mathrm{curl}\;\mathrm{curl}\vec{E}+\mu_{0}\frac{\partial^{2}{\vec{D}}^{L}}{\partial t^{2}}=-\mu_{0}\frac{\partial^{2}{\vec{D}}^{NL}}{\partial t^{2}}$$

where $\mu_{0}$ is the free space permeability, ${\vec{D}}^{L}$ and ${\vec{D}}^{NL}$ are the linear and nonlinear parts of the electric induction, respectively.

The SLS in the liquid crystalline waveguide with a SALC core is described by the coupled wave equations of the type (1) for the waveguide modes and the hydrodynamic equations for SALC. The SALC hydrodynamics in general case is very complicated taking into account the anisotropy and including the fluctuations of the mass density ρ, the layer displacement $u\left(\vec{r},t\right)$ along the *Z* axis normal to the layers and the change of the director $\vec{n}$ [15,16]. The character of the fluctuation modes is determined by the propagation direction [15,16,17,18]. We assume that the SALC temperature is far from the temperature $T_{SmA-N}$ of the SALC-NLC phase transition. Since the optical losses in SALC are negligible [2] the waveguide temperature is assumed to be constant and the smectic A phase is stable. In such a case, the system of hydrodynamic equations for SALC has the form [15].

(2) $$\rho \frac{\partial v_{i}}{\partial t}=-\frac{\partial \Pi }{\partial x_{i}}+\Lambda_{i}+\frac{\partial \sigma_{ik}^{\prime }}{\partial x_{k}}$$

(3) $$\Lambda_{i}=-\frac{\delta F}{\delta u_{i}}$$

(4) $$\sigma_{ik}^{\prime }=\alpha_{0}\delta_{ik}A_{ll}+\alpha_{1}\delta_{iz}A_{zz}+\alpha_{4}A_{ik}+\alpha_{56}\left(\delta_{iz}A_{zk}+\delta_{kz}A_{zi}\right)+\alpha_{7}\delta_{iz}\delta_{kz}A_{ll}$$

(5) $$A_{ik}=\frac{1}{2}\left(\frac{\partial v_{i}}{\partial x_{k}}+\frac{\partial v_{k}}{\partial x_{i}}\right)$$

(6) $$\mathrm{div}\vec{v}=0$$

(7) $$v_{z}=\frac{\partial u}{\partial t}$$

(8) $$\delta_{ik}=\left\{\begin{array}{cc} 1, & i=k\\ 0, & i\neq k \end{array}\right.$$

where $\vec{v}$ is the hydrodynamic velocity, Π is the pressure, $\vec{\Lambda }$ is the generalized force density, $\sigma_{ik}^{\prime }$ is the viscous stress tensor, $\alpha_{i}$ are the viscosity Leslie coefficients, *F* is the free energy density of SALC. The SALC free energy density in the presence of the external electric field $\vec{E}\left(\vec{r},t\right)$ has the form.

(9) $$F=\frac{1}{2}B{\left(\frac{\partial u}{\partial z}\right)}^{2}+\frac{1}{2}K{\left(\frac{\partial^{2}u}{\partial x^{2}}+\frac{\partial^{2}u}{\partial y^{2}}\right)}^{2}-\frac{1}{2}\varepsilon_{0}\varepsilon_{ik}E_{i}E_{k}$$

Here $B∼10^{6}-10^{7}$ J/m${}^{3}$ is the elastic constant related to the layer compression, $K∼10^{-11}$ N is the Frank elastic constant associated with the SALC purely orientational energy, $\varepsilon_{0}$ is the free space permittivity, and $\varepsilon_{ik}$ is the SALC permittivity tensor including the nonlinear terms related to the smectic layer strains. SALC is an optically uniaxial medium with the optical axis *Z* normal to the layer plane. It is given by [16].

(10) $$\varepsilon_{xx}=\varepsilon_{yy}=\varepsilon_{⊥}+a_{⊥}\frac{\partial u}{\partial z}$$

(11) $$\varepsilon_{zz}=\varepsilon_{\|}+a_{\|}\frac{\partial u}{\partial z}$$

(12) $$\varepsilon_{xz}=\varepsilon_{zx}=-\varepsilon_{a}\frac{\partial u}{\partial x},\varepsilon_{yz}=\varepsilon_{zy}=-\varepsilon_{a}\frac{\partial u}{\partial y}$$

where $\varepsilon_{⊥},\varepsilon_{\|}$ are the diagonal components of the permittivity tensor perpendicular and parallel to the optical axis, respectively, $a_{⊥}∼1,a_{\|}∼1$ are the phenomenological dimensionless coefficients, and $\varepsilon_{a}$ is the permittivity anisotropy. In our case, the losses in SALC can be neglected and the linear permittivity is real [2].

(13) $$\varepsilon_{a}=\varepsilon_{\|}-\varepsilon_{⊥}$$

For the wave vector ${\vec{k}}_{S}$ oblique to the smectic layer plane in SALC there exist two practically uncoupled acoustic modes. One of these modes is the ordinary longitudinal sound wave caused by the mass density oscillations and described by the dispersion relation $\Omega =s_{1}k_{S}$ independent of the propagation direction where the sound velocity $s_{1}=\sqrt{A/\rho }$, and *A* is the elastic constant related to bulk compression [15,16,17,18]. The second mode is the so-called second sound (SS) with the following dispersion relation [15,17].

(14) $$\Omega_{SS}=s_{2}\frac{k_{S⊥}k_{Sz}}{k_{S}},\;s_{2}=\sqrt{\frac{B}{\rho }}$$

where $s_{2}$ is the SS velocity, $k_{S⊥}$, $k_{Sz}$ are the SS wave vector components in the layer plane and normal to it, respectively. SS corresponds to the changes in the layer spacing, it is neither longitudinal, nor transverse, and vanishes for the wave vector parallel or perpendicular to the smectic layer plane as it is seen from Equation (14) [15,16,17,18]. Since the elastic constant $B\ll A∼10^{9}$ J/m${}^{3}$, the SS may propagate in the SALC without the density change [15,16,17,18]. SS has been observed experimentally [19,20,21]. In such a case, SALC may be considered to be incompressible liquid according to Equation (6), the pressure Π=0, and the SALC energy density *F* determined by Equation (9) does not include the bulk compression term. The purely orientational term second term in Equation (9) can be neglected since for the typical values of the elastic constants and $k_{S}∼10^{5}$ m${}^{-1}$ $B\gg Kk_{S}^{2}$. The normal layer displacement $u\left(\vec{r},t\right)$ by definition has only one component along the *Z* axis. Hence, the generalized force density $\vec{\Lambda }$ has only the *z* component according to Equation (3): $\vec{\Lambda }=\left(0,0,\Lambda_{z}\right)$. Equation (7) is specific for SALC since it determines the condition of the smectic layer continuity and the absence of the permeation process which can be neglected in the high frequency limit [15,17]. Taking into account the assumptions mentioned above and combining Equations (2)–(12) we obtain the equation of motion for smectic layer normal displacement $u\left(\vec{r},t\right)$ in an external electric field $\vec{E}\left(\vec{r},t\right)$ [36].

$$-\rho \nabla^{2}\frac{\partial^{2}u}{\partial t^{2}}+\left[\alpha_{1}\nabla_{⊥}^{2}\frac{\partial^{2}}{\partial z^{2}}+\frac{1}{2}\left(\alpha_{4}+\alpha_{56}\right)\nabla^{2}\nabla^{2}\right]\frac{\partial u}{\partial t}+B\nabla_{⊥}^{2}\frac{\partial^{2}u}{\partial z^{2}}$$

(15) $$=\frac{\varepsilon_{0}}{2}\nabla_{⊥}^{2}\left\{-2\varepsilon_{a}\left[\frac{\partial }{\partial x}\left(E_{x}E_{z}\right)+\frac{\partial }{\partial y}\left(E_{y}E_{z}\right)\right]+\frac{\partial }{\partial z}\left[a_{⊥}\left(E_{x}^{2}+E_{y}^{2}\right)+a_{\|}E_{z}^{2}\right]\right\}$$

where $\nabla_{⊥}^{2}=\left(\partial^{2}/\partial x^{2}\right)+\left(\partial^{2}/\partial y^{2}\right)$. Taking into account the SALC symmetry we may choose without the loss of generality the propagation plane in a slab waveguide as the xz plane. Then, using expressions (10)–(12) we obtain for the linear and nonlinear parts of the electric induction ${\vec{D}}^{L}$ and ${\vec{D}}^{NL}$.

(16) $$D_{x,y}^{L}=\varepsilon_{0}\varepsilon_{⊥}E_{x,y},D_{z}^{L}=\varepsilon_{0}\varepsilon_{\|}E_{z}$$

(17) $$D_{x}^{NL}=\varepsilon_{0}\left(a_{⊥}\frac{\partial u}{\partial z}E_{x}-\varepsilon_{a}\frac{\partial u}{\partial x}E_{z}\right);D_{y}^{NL}=\varepsilon_{0}a_{⊥}\frac{\partial u}{\partial z}E_{y}$$

(18) $$D_{z}^{NL}=\varepsilon_{0}\left(a_{\|}\frac{\partial u}{\partial z}E_{z}-\varepsilon_{a}\frac{\partial u}{\partial x}E_{x}\right)$$

It is seen from Equations (17) and (18) that the nonlinear polarization in SALC is related to the smectic layer normal and tangential strain ∂u/∂z and ∂u/∂x as it was mentioned above [26,27,28,29,30,31,32,33,34,35,36]. We solve the wave Equation (1) according to the SVA approximation procedure [37]. In the linear approximation, we solve the homogeneous part of Equation (1) neglecting the nonlinear polarization (17) and (18).

(19) $$\mathrm{curl}\;\mathrm{curl}\vec{E}+\mu_{0}\frac{\partial^{2}{\vec{D}}^{L}}{\partial t^{2}}=0$$

We obtain from Equation (19) the general solution and the linear dispersion relations for the waveguide modes [38,39]. Then, we evaluate the nonlinear polarization (17) and (18), derive the truncated equations for the SVAs of the waveguide mode electric fields in the SALC core and evaluate the complex SVA magnitudes and phases [37,38]. In the next section, we evaluate the waveguide modes and the nonlinear polarization defined by Equations (17) and (18).

## 3. Nonlinear Polarization in the SALC Core of the Waveguide

The TM and TE mode electric and magnetic fields have the form, respectively [38,39,40].

(20) $${\vec{H}}_{TM}\left(x,z,t\right)=H_{y}\left(x,z,t\right)a_{y};\;{\vec{E}}_{TM}\left(x,z,t\right)=\left(E_{x}\left(x,z,t\right),0,E_{z}\left(x,z,t\right)\right)$$

(21) $${\vec{E}}_{TE}\left(x,z,t\right)=E_{y}\left(x,z,t\right)a_{y};\;{\vec{H}}_{TE}\left(x,z,t\right)=\left(H_{x}\left(x,z,t\right),0,H_{z}\left(x,z,t\right)\right)$$

We consider separately the TM and TE modes propagating in the slab optical waveguide with the SALC core because Equations (15)–(18) show that in the framework of the slab waveguide model TE and TM modes do not interact. We start with the analysis of the TM even modes. Assuming that the waveguide is symmetric with the identical claddings z>d, z<−d characterized by the same permittivity $\varepsilon_{r2}$ and the refraction index $n_{2}=\sqrt{\varepsilon_{r2}}$, solving Equation (1) in the linear approximation and using the boundary conditions for the tangential components of the magnetic and electric field in the cladding $H_{yC}$ and $E_{xC}$ and in the SALC core $H_{ySA}$ and $E_{xSA}$, respectively [38,39,40].

(22) $$H_{yC}\left(z=d\right)=H_{ySA}\left(z=d\right);E_{xC}\left(z=d\right)=E_{xSA}\left(z=d\right)$$

we obtain for the electric field $E_{x,zSA}$, $E_{x,zC}$ in the SALC core $\left|z\right|\leq d$ and in the cladding z>d, z<−d, respectively [35,39].

(23) $$E_{xSA}=-iE_{0zSA}\frac{k\varepsilon_{∥}}{\beta \varepsilon_{⊥}}\sin kz\exp\left[i\left(\omega t-\beta x\right)\right]$$

(24) $$E_{zSA}=-E_{0zSA}\cos kz\exp\left[i\left(\omega t-\beta x\right)\right]$$

(25) $$E_{xC}=\left\{\begin{array}{c} i\frac{\alpha }{\beta }E_{0zC}\exp\left(-\alpha z\right)\exp i\left(\omega t-\beta x\right),\;z>d\\ -i\frac{\alpha }{\beta }E_{0zC}\exp\left(\alpha z\right)\exp i\left(\omega t-\beta x\right),\;z<-d \end{array}\right.$$

(26) $$E_{zC}=\left\{\begin{array}{c} E_{0zC}\exp\left(-\alpha z\right)\exp i\left(\omega t-\beta x\right),\;z>d\\ E_{0zC}\exp\left(\alpha z\right)\exp i\left(\omega t-\beta x\right),\;z<-d \end{array}\right.$$

Here ω is the optical mode angular frequency, β is the propagation constant, *k* is the wave vector in the core, and α is the wavenumber in the cladding. They are given by

(27) $$\beta =\sqrt{\varepsilon_{∥}\left[{\left(\frac{\omega }{c}\right)}^{2}-\frac{k^{2}}{\varepsilon_{⊥}}\right]}$$

(28) $$\alpha =\sqrt{\beta^{2}-\frac{\omega^{2}}{c^{2}}\varepsilon_{r2}}$$

Expression (27) shows that the TM mode propagates in an anisotropic medium as an extraordinary wave [41]. The wave vector *k* for the TM even modes is defined by the dispersion relation

(29) $$\tan kd=\frac{\varepsilon_{⊥}}{\varepsilon_{r2}}\sqrt{{\left(\frac{V}{kd}\right)}^{2}-\frac{\varepsilon_{∥}}{\varepsilon_{⊥}}};V=\frac{2\pi d}{\lambda_{0}}\sqrt{\varepsilon_{∥}-\varepsilon_{r2}};\varepsilon_{∥}>\varepsilon_{r2}$$

where $\lambda_{0}=2\pi c/\omega$ and *c* are the free space wavelength and light velocity, respectively. Consider now the TM odd modes. In this case, the electric field components $E_{x,zSA}^{odd}$ in the SALC has the form [39].

(30) $$E_{zSA}^{odd}=E_{0zSA}^{odd}\sin kz\exp i\left(\omega t-\beta x\right)\;$$

(31) $$E_{xSA}^{odd}=-\frac{ik\varepsilon_{∥}}{\beta \varepsilon_{⊥}}E_{0zSA}^{odd}\cos kz\exp i\left(\omega t-\beta x\right)$$

The boundary conditions (22) give the following dispersion relation for the TM odd modes.

(32) $$-\cot kd=\frac{\varepsilon_{⊥}}{\varepsilon_{r2}}\sqrt{{\left(\frac{V}{kd}\right)}^{2}-\frac{\varepsilon_{∥}}{\varepsilon_{⊥}}}$$

**Figure 2:**
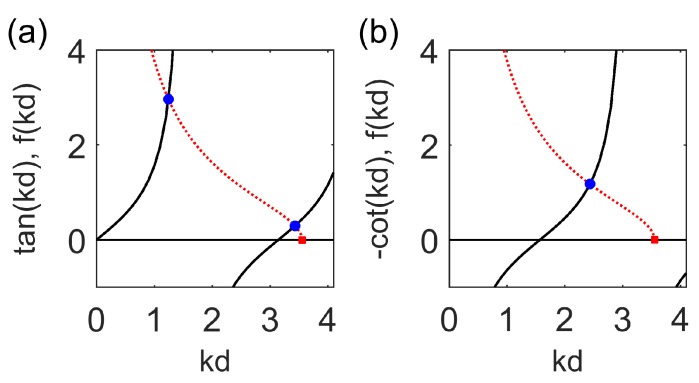
Graphic solution of the disperison relations for the TM even modes (**a**) and odd modes (**b**); $f\left(kd\right)=\frac{\varepsilon_{⊥}}{\varepsilon_{r2}}\sqrt{{\left(\frac{V}{kd}\right)}^{2}-\frac{\varepsilon_{∥}}{\varepsilon_{⊥}}}$, $\omega =5\pi \times 10^{14}s^{-1}$.

**Figure 3:**
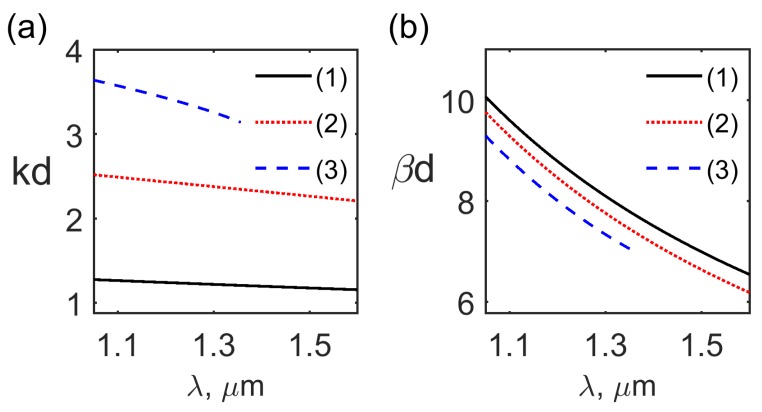
The normalized wavenumber kd (**a**) and propagation constant βd (**b**) dependence on the optical wavelength λ for the even modes TM${}_{0,1}^{even}$ (curves 1, 3) and the odd mode TM${}_{1}^{odd}$ (curve 2).

**Figure 4:**
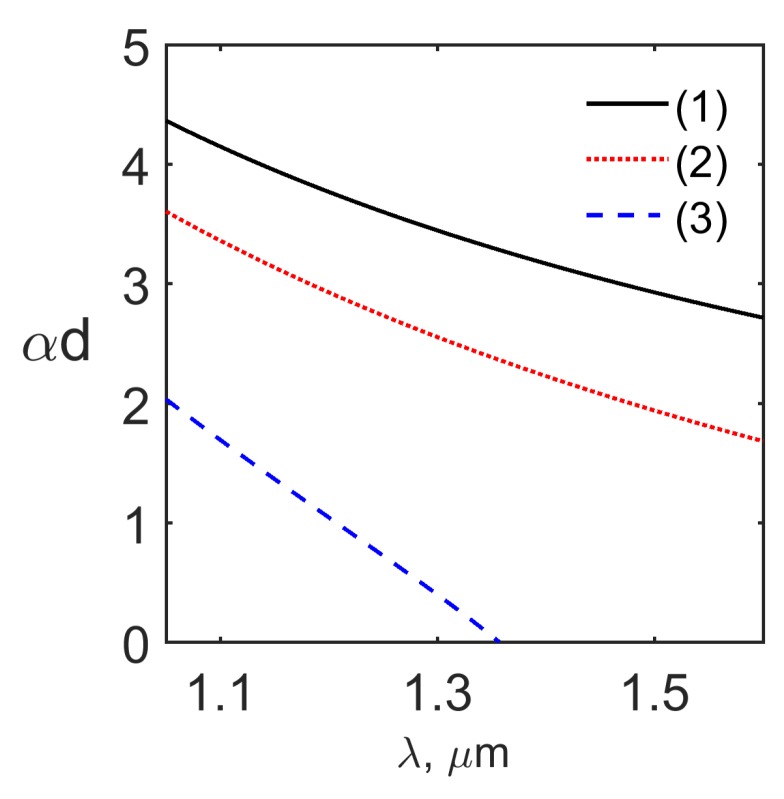
The dependence of normalized wavenumber in the cladding αd for the even modes TM${}_{0,1}^{even}$ (curves 1, 3) and odd mode TM${}_{1}^{odd}$ (curve 2) on the optical wavelength.

The solution of the dispersion relations (29) and (32) for the TM even and odd modes are presented in Figure 2a,b, respectively. It is seen from Figure 2a,b that for the frequency $\omega =5\pi \times 10^{14}$ s${}^{-1}$ and for the typical values of the waveguide parameters there exist two even TM modes TM${}_{0,1}^{even}$ and one odd TM mode TM${}_{1}^{odd}$. The normalized wavenumber kd and propagation constant βd dependence on the optical wavelength λ for the even modes TM${}_{0,1}^{even}$ and for the odd mode TM${}_{1}^{odd}$ are presented in Figure 3a,b, respectively. The normalized wavenumber in the cladding αd spectral dependence is shown in Figure 4. It is seen from Figure 4 that the fundamental even mode TM${}_{0}^{even}$ does not have a cutoff while the second even mode TM${}_{1}^{even}$ has a cutoff wavelength coinciding with the cutoff wavelength in Figure 3a,b, respectively. Comparison of Figure 3a and Figure 4 shows that in the wavelength region under consideration kd>π/2, and αd≠0 for the odd mode TM${}_{1}^{odd}$ [39]. The solutions of the dispersion relations (29) and (32) presented in Figure 3a,b show that for the waveguide SALC core thickness of 2d=2 μm, the typical values of LC and cladding permittivity [8], and the wavelengths $\lambda_{0}\approx 1.4$–1.55 μm important for optical communications the single mode regime occurs. We consider the interaction of the TM modes with the close optical frequencies $\omega_{1,2}$ such that the frequency shift $\Delta \omega =\omega_{1}-\omega_{2}∼10^{8}$–$10^{9}$ s${}^{-1}\ll \omega_{1}$ which is typical for the light scattering in SALC [15,19]. The numerical estimations of the propagation constant β and the wave vector *k* according to Equations (27) and (29) show that for the frequency shifts $\Delta \omega ∼10^{8}$–$10^{9}$ s${}^{-1}$ the values of β and *k* are practically the same for the TM modes with the close frequencies $\omega_{1,2}$. Consequently, the strong interaction occurs only for the counter-propagating TM modes. For the sake of definiteness, we consider the interaction of the TM even modes (23) and (24). Obviously, the nonlinear interaction of the TM odd modes would be practically the same. Using expressions (23) and (24) we can write for such TM even mode electric field [35].

(33) $${\vec{E}}_{SA1,2}=\frac{1}{2}E_{0zSA1,2}\left(x,t\right)\left\{-{\vec{a}}_{x}i\frac{k\varepsilon_{∥}}{\beta \varepsilon_{⊥}}\sin kz\mp {\vec{a}}_{z}\cos kz\right\}\exp\left[i\left(\omega_{1,2}t\mp \beta x\right)\right]+c.c.$$

where c.c. stands for complex conjugate, and ${\vec{a}}_{x}$, ${\vec{a}}_{z}$ are the unit vectors of the *X* and *Z* axes, respectively. We assume that $E_{0zSA1,2}\left(x,t\right)=\left|E_{0zSA1,2}\right|\exp i\theta_{1,2}$ are the complex SVAs [37].

(34) $$\left|\frac{\partial^{2}E_{0zSA1,2}}{\partial x^{2}}\right|\ll \left|\beta \frac{\partial E_{0zSA1,2}}{\partial x}\right|;\left|\frac{\partial^{2}E_{0zSA1,2}}{\partial t^{2}}\right|\ll \left|\omega \frac{\partial E_{0zSA1,2}}{\partial t}\right|$$

At the small distances of several mm typical for the optical waveguide length the dependence of SVAs on *x* and the dispersion effects can be neglected, and the SVAs $E_{0zSA1,2}\left(t\right)$ depend only on time. Substituting expressions (33) into equation of motion (15) and keeping in the right-hand side (RHS) only the terms with the frequency difference Δω we obtain.

(35) $$\begin{array}{c} -\rho \nabla^{2}\frac{\partial^{2}u}{\partial t^{2}}+\left[\alpha_{1}\frac{\partial^{2}}{\partial x^{2}}\frac{\partial^{2}}{\partial z^{2}}+\frac{1}{2}\left(\alpha_{4}+\alpha_{56}\right)\nabla^{2}\nabla^{2}\right]\frac{\partial u}{\partial t}+B\frac{\partial^{2}}{\partial x^{2}}\frac{\partial^{2}u}{\partial z^{2}}\\ =-2\varepsilon_{0}\beta^{2}kE_{0zSA1}E_{0zSA2}^{*}\left[\varepsilon_{a}\frac{\varepsilon_{∥}}{\varepsilon_{⊥}}+a_{⊥}\frac{1}{2}{\left(\frac{k\varepsilon_{∥}}{\beta \varepsilon_{⊥}}\right)}^{2}+\frac{1}{2}a_{\|}\right]\\ \times \sin2kz\exp\left\{i\left[\left(\omega_{1}-\omega_{2}\right)t-2\beta x\right]\right\}+c.c. \end{array}$$

Then the particular solution of Equation (35) related to its RHS yields the dynamic grating of the smectic layer normal displacement.

(36) $$u\left(x,z,t\right)=U_{0}\sin2kz\exp\left\{i\left[\left(\omega_{1}-\omega_{2}\right)t-2\beta x\right]\right\}+c.c.$$

where

(37) $$U_{0}=\frac{\varepsilon_{0}\beta^{2}kE_{0zSA1}E_{0zSA2}^{*}\left[\varepsilon_{a}\frac{\varepsilon_{∥}}{\varepsilon_{⊥}}+a_{⊥}\frac{1}{2}{\left(\frac{k\varepsilon_{∥}}{\beta \varepsilon_{⊥}}\right)}^{2}+\frac{1}{2}a_{\|}\right]}{2\rho \left(\beta^{2}+k^{2}\right)G\left(k,\beta ,\Delta \omega \right)}$$

(38) $$G\left(k,\beta ,\Delta \omega \right)={\left(\Delta \omega \right)}^{2}-i\Delta \omega \Gamma -\Omega^{2}$$

(39) $$\Gamma =\frac{1}{\rho }\left[4\frac{\alpha_{1}\beta^{2}k^{2}}{\left(\beta^{2}+k^{2}\right)}+2\left(\alpha_{4}+\alpha_{56}\right)\left(\beta^{2}+k^{2}\right)\right];\;\Omega^{2}=4\frac{B\beta^{2}k^{2}}{\rho \left(\beta^{2}+k^{2}\right)}$$

**Figure 5:**
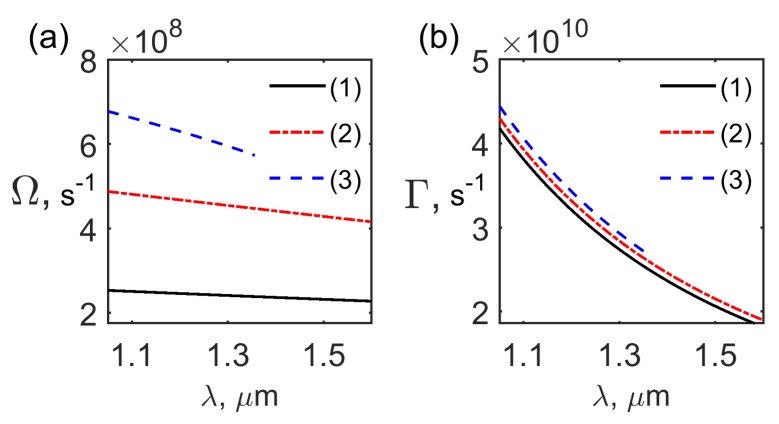
The SS frequency Ω (**a**) and decay constant Γ (**b**) dependence on the optical wavelength for the even modes TM${}_{0,1}^{even}$ (curves 1,3) and the odd mode TM${}_{1}^{odd}$ (curve 2).

Here Ω, Γ are SS frequency and decay factor, respectively [15,16,17,18,19,20,21]. The SS frequency Ω and decay factor Γ dependence on the optical wavelength λ for the first two TM modes are presented in Figure 5a,b, respectively. Numerical estimations show that for the typical values of SALC parameters [15,16,17,18,19,20,21], the optical wavelength in the range of $\lambda_{opt}∼1.3$–1.55 μm and $\Delta \omega ∼10^{8}$–$10^{9}$ s${}^{-1}$ the homogeneous layer oscillations are overdamped. For this reason, the rapidly decaying homogeneous solution of Equation (35) can be neglected. We have taken into account only the solution (36) enhanced by the interfering optical TM modes (33).

Substituting expressions (33) and (36) into Equations (17) and (18) we evaluate the nonlinear part of the electric induction ${\vec{D}}^{NL}=\left(D_{x}^{NL},0,D_{z}^{NL}\right)$ which has only *x* and *z* components for the TM modes. Separating the phase matched parts of ${\vec{D}}^{NL}$ with the frequencies $\omega_{1,2}$, respectively, we obtain.

(40) $$\begin{array}{c} D_{x}^{NL}\left(\omega_{1}\right)=\varepsilon_{0}U_{0}i\beta E_{0zSA2}\sin kz\exp\left[i\left(\omega_{1}t-\beta x\right)\right]\\ \times \left\{-a_{⊥}\frac{k^{2}\varepsilon_{∥}}{\beta^{2}\varepsilon_{⊥}}\cos2kz+2\varepsilon_{a}\cos^{2}kz\right\} \end{array}$$

(41) $$\begin{array}{c} D_{z}^{NL}\left(\omega_{1}\right)=\varepsilon_{0}kU_{0}E_{0zSA2}\cos kz\exp\left[i\left(\omega_{1}t-\beta x\right)\right]\\ \times \left\{a_{\|}\cos2kz+2\varepsilon_{a}\frac{\varepsilon_{∥}}{\varepsilon_{⊥}}\sin^{2}kz\right\} \end{array}$$

(42) $$\begin{array}{c} D_{x}^{NL}\left(\omega_{2}\right)=\varepsilon_{0}U_{0}^{*}iE_{0zSA1}\sin kz\exp\left[i\left(\omega_{2}t+\beta x\right)\right]\\ \times \left\{-a_{⊥}\frac{k^{2}\varepsilon_{∥}}{\beta \varepsilon_{⊥}}\cos2kz+2\varepsilon_{a}\beta \cos^{2}kz\right\} \end{array}$$

(43) $$\begin{array}{c} D_{z}^{NL}\left(\omega_{2}\right)=-\varepsilon_{0}kU_{0}^{*}E_{0zSA1}\cos kz\exp\left[i\left(\omega_{2}t+\beta x\right)\right]\\ \times \left\{a_{\|}\cos2kz+2\varepsilon_{a}\frac{\varepsilon_{∥}}{\varepsilon_{⊥}}\sin^{2}kz\right\} \end{array}$$

The nonlinear polarization (40)–(43) is related to the specific cubic nonlinearity related to the smectic layer displacement which occurs without the change of the SALC mass density.

The electric field $E_{0ySA1,2}$ of the TE modes (21) is perpendicular to the optical axis *Z*. It has the form.

$${\vec{E}}_{SA1,2}=\frac{1}{2}E_{0ySA1,2}\left(x,t\right){\vec{a}}_{y}\cos kz\exp\left[i\left(\omega_{1,2}t\mp \beta x\right)\right]+c.c.$$

TE modes propagate in an anisotropic medium as ordinary waves with the propagation constant $\beta^{2}=\left(\omega^{2}\varepsilon_{⊥}/c^{2}\right)-k^{2}$ including only the transverse permittivity $\varepsilon_{⊥}$ [41]. The boundary conditions for the TE modes have the form.

(44) $$E_{yC}\left(d\right)=E_{ySA}\left(d\right);H_{zC}\left(d\right)=H_{zSA}\left(d\right)$$

They yield the TE mode dispersion relation similar to the isotropic medium [38].

(45) $$\tan kd=\sqrt{\frac{V_{TE}^{2}}{{\left(kd\right)}^{2}}-1};\;V=\frac{2\pi d}{\lambda_{0}}\sqrt{\varepsilon_{⊥}-\varepsilon_{r2}},\;\varepsilon_{⊥}>\varepsilon_{r2}$$

In LC typically $\varepsilon_{∥}>\varepsilon_{⊥}$ [2,14], and under the condition $\varepsilon_{∥}>\varepsilon_{r2}>\varepsilon_{⊥}$ only TM modes can propagate in the slab optical waveguide. In general case, the nonlinear polarization enhanced by the TE modes includes only the component $D_{y}^{NL}=\varepsilon_{0}a_{⊥}\left(\partial u/\partial z\right)E_{y}$ as it is seen from expression (17), and the dynamic grating amplitude $U_{0TE}∼E_{0ySA1}E_{0ySA2}^{*}$. Obviously, the nonlinear interaction of the TE modes is isotropic and less pronounced than the TM mode interaction including both the longitudinal and the transverse component of the electric field. For this reason, we analyze in detail the TM mode nonlinear interaction.

## 4. Evaluation of the TM Mode SVAs

Using the standard procedure [37], we substitute expressions (33), (16), and (40)–(43) into Equation (1), separate the linear and nonlinear parts, neglect the small terms ∼$\left|\partial^{2}E_{0zSA1,2}/\partial t^{2}\right|$ according to the SVA approximation condition (34) and equate the phase matched terms the frequencies $\omega_{1,2}$, respectively. Then we obtain the coupled equations for the SVAs $E_{0zSA1,2}\left(t\right)$.

(46) $$\begin{array}{c} \varepsilon_{\|}\frac{\partial E_{0zSA1}}{\partial t}\left\{a_{x}\frac{k}{\beta }\sin kz-a_{z}i\cos kz\right\}\\ =\omega_{1}U_{0}E_{0zSA2}\{a_{x}i\beta \sin kz\left[-a_{⊥}\frac{k^{2}\varepsilon_{∥}}{\beta^{2}\varepsilon_{⊥}}\cos2kz+2\varepsilon_{a}\cos^{2}kz\right]\\ +a_{z}k\cos kz\left[a_{\|}\cos2kz+2\varepsilon_{a}\frac{\varepsilon_{∥}}{\varepsilon_{⊥}}\sin^{2}kz\right]\} \end{array}$$

(47) $$\begin{array}{c} \varepsilon_{\|}\frac{\partial E_{0zSA2}}{\partial t}\left\{a_{x}\frac{k}{\beta }\sin kz+a_{z}i\cos kz\right\}\\ =\omega_{2}U_{0}^{*}E_{0zSA1}\{a_{x}i\beta \sin kz\left[-a_{⊥}\frac{k^{2}\varepsilon_{∥}}{\beta^{2}\varepsilon_{⊥}}\cos2kz+2\varepsilon_{a}\cos^{2}kz\right]\\ -a_{z}k\cos kz\left[a_{\|}\cos2kz+2\varepsilon_{a}\frac{\varepsilon_{∥}}{\varepsilon_{⊥}}\sin^{2}kz\right]\} \end{array}$$

We multiply Equations (46) and (47) by the vectors ${\left\{a_{x}\frac{k}{\beta }\sin kz-a_{z}i\cos kz\right\}}^{*}$ and ${\left\{a_{x}\frac{k}{\beta }\sin kz+a_{z}i\cos kz\right\}}^{*}$, respectively, substitute the SVA expressions

(48) $$E_{0zSA1,2}\left(x,t\right)=\left|E_{0zSA1,2}\right|\exp i\theta_{1,2}$$

and separate the real and imaginary parts of the resulting equations. Then we obtain the following equations for the magnitudes $\left|E_{0zSA1,2}\right|$ and phases $\theta_{1,2}$ of the TM mode SVAs.

(49) 1ω1∂E0zSA12∂tF1z=ε0E0zSA12E0zSA22β2k2hImGk,β,Δωε‖ρβ2+k2Gk,β,Δω2F2z

(50) 1ω2∂E0zSA22∂tF1z=−ε0E0zSA12E0zSA22β2k2hImGk,β,Δωε‖ρβ2+k2Gk,β,Δω2F2z

(51) ε‖∂θ1∂tF1z=ω1ε0E0zSA22β2k2hReGk,β,Δω2ρβ2+k2Gk,β,Δω2F2z

(52) ε‖∂θ2∂tF1z=ω2ε0β2k2E0zSA12hReGk,β,Δω2ρβ2+k2Gk,β,Δω2F2z

where

(53) $$\begin{array}{c} F_{1}\left(z\right)={\left(\frac{k}{\beta }\right)}^{2}\sin^{2}kz+\cos^{2}kz;\;\;F_{2}\left(z\right)=\{\sin^{2}kz\left[-a_{⊥}\frac{k^{2}\varepsilon_{∥}}{\beta^{2}\varepsilon_{⊥}}\cos2kz+2\varepsilon_{a}\cos^{2}kz\right]\\ +\cos^{2}kz\left[a_{\|}\cos2kz+2\varepsilon_{a}\frac{\varepsilon_{∥}}{\varepsilon_{⊥}}\sin^{2}kz\right]\} \end{array}$$

and

(54) $$h=\varepsilon_{a}\frac{\varepsilon_{∥}}{\varepsilon_{⊥}}+a_{⊥}\frac{1}{2}{\left(\frac{k\varepsilon_{∥}}{\beta \varepsilon_{⊥}}\right)}^{2}+\frac{1}{2}a_{\|}$$

Combining Equations (49) and (50) we obtain for the SVA magnitudes $\left|E_{0zSA1,2}\right|$

(55) $$\frac{\partial }{\partial t}\left(\frac{{\left|E_{0zSA1}\right|}^{2}}{\omega_{1}}+\frac{{\left|E_{0zSA2}\right|}^{2}}{\omega_{2}}\right)=0$$

and

(56) $$\frac{{\left|E_{0zSA1}\right|}^{2}}{\omega_{1}}+\frac{{\left|E_{0zSA2}\right|}^{2}}{\omega_{2}}=const=I_{0}$$

where

(57) $$I_{0}=\frac{{\left|E_{0zSA1}\left(0\right)\right|}^{2}}{\omega_{1}}+\frac{{\left|E_{0zSA2}\left(0\right)\right|}^{2}}{\omega_{2}}$$

Equation (56) is the Manley-Rowe relation for the SVA magnitudes $\left|E_{0zSA1,2}\right|$ which corresponds to the conservation of the photon number in the SLS process [37]. It is seen from Equation (38) that for $\Delta \omega =\omega_{1}-\omega_{2}>0$ the imaginary part ImGk,β,Δω<0, and the intensity ${\left|E_{0zSA1}\right|}^{2}$ of the TM mode with the higher frequency $\omega_{1}$ is decreasing with time while the intensity ${\left|E_{0zSA2}\right|}^{2}$ of the TM mode with the lower frequency $\omega_{2}$ is increasing. Consequently, the TM modes with the frequencies $\omega_{1,2}$ are the pumping and signal waves, respectively, and the Stokes type SLS occurs [37]. Equations (49) and (50) describe the energy exchange between the TM modes, while Equations (51) and (52) describe the cross-phase modulation (XPM) process.

We introduce the dimensionless variables

(58) $$I_{1,2}=\frac{{\left|E_{0zSA1,2}\right|}^{2}}{\omega_{1,2}I_{0}}$$

such that $I_{1}+I_{2}=1$. Substituting expressions (58) into Equations (49) and (50), integrating both parts of these equations over *z* from −d up to *d* and using the Manley-Rowe relation (56) we obtain the following solutions for the normalized SVA intensities $I_{1,2}$.

(59) $$I_{1}\left(t\right)=\frac{I_{1}\left(0\right)}{\left\{\left(1-I_{1}\left(0\right)\right)\exp\left[gF\left(kd\right)t\right]+I_{1}\left(0\right)\right\}}$$

(60) $$I_{2}\left(t\right)=\frac{\left(1-I_{1}\left(0\right)\right)}{\left(1-I_{1}\left(0\right)\right)+I_{1}\left(0\right)\exp\left[-gF\left(kd\right)t\right]}$$

where the gain *g* and the geometric factor $F\left(kd\right)$ are given by.

(61) g=ε0ε‖ω1ω2I0β2k2hImGk,β,Δωρβ2+k2Gk,β,Δω2>0

(62) $$\begin{array}{c} F\left(kd\right)=\{\left[a_{⊥}\frac{k^{2}\varepsilon_{∥}}{\beta^{2}\varepsilon_{⊥}}+\varepsilon_{a}\left(1+\frac{\varepsilon_{∥}}{\varepsilon_{⊥}}\right)+a_{\|}\right]kd+\left[-a_{⊥}\frac{k^{2}\varepsilon_{∥}}{\beta^{2}\varepsilon_{⊥}}+a_{\|}\right]\sin2kd\\ +\frac{1}{4}\left[a_{⊥}\frac{k^{2}\varepsilon_{∥}}{\beta^{2}\varepsilon_{⊥}}-\varepsilon_{a}\left(1+\frac{\varepsilon_{∥}}{\varepsilon_{⊥}}\right)+a_{\|}\right]\sin4kd\}{\left\{\left[{\left(\frac{k}{\beta }\right)}^{2}+1\right]2kd+\left[1-{\left(\frac{k}{\beta }\right)}^{2}\right]\sin2kd\right\}}^{-1} \end{array}$$

The pumping intensity threshold in the SLS process described by expressions (59) and (60) is absent since the losses in SALC can be neglected as it was mentioned above. Comparison of expressions (59) and (60) shows that for the initial pumping wave intensity larger than the initial signal wave intensity $I_{1}\left(0\right)>I_{2}\left(0\right)$ the crossing time $t_{0}>0$ exists where $I_{1}\left(t_{0}\right)=I_{2}\left(t_{0}\right)$. It is given by.

(63) $$t_{0}=\frac{1}{gF\left(kd\right)}\ln\left[\frac{I_{1}\left(0\right)}{I_{2}\left(0\right)}\right]$$

Substituting expression (63) into expressions (59) and (60) we obtain.

(64) $$I_{1,2}\left(t\right)=\frac{1}{2}\left\{1\mp \tanh\left[\frac{1}{2}gF\left(kd\right)\left(t-t_{0}\right)\right]\right\}$$

**Figure 6:**
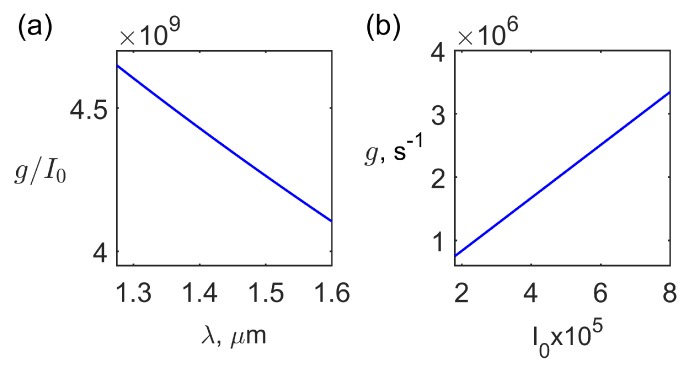
The normalized gain $g/I_{0}$ ( s${}^{-2}$V${}^{-2}$m${}^{2}$) dependence on the optical wavelength λ (**a**); the gain *g* dependence on the normalized intensity $I_{0}$ (V${}^{2}$m${}^{-2}$s) for the optical wavelength λ=1.55 μm (**b**).

The spectral dependence of the gain *g* and its dependence on the normalized intensity $I_{0}$ are presented in Figure 6a,b.

Figure 6a shows that the gain is slightly varying in the optical wavelength range of interest because Γ≫Ω as it is seen from Figure 5a,b. The gain *g* has a maximum value $g_{\max}$ at the SS resonance condition when Δω=Ω and ReGk,β,Δω=0 according to expression (38). The numerical estimations show that for the typical values of $k,\beta ∼10^{6}$ m${}^{-1}$ and $\Delta \omega ∼10^{8}-10^{9}$ s${}^{-1}$ the SS resonance condition can be satisfied. The numerical estimations also show that for the values of kd defined by the dispersion relation (29) $F\left(kd\right)∼1$. The dependence of the gain *g* on the normalized intensity $I_{0}$ is linear as it is seen from Figure 6b. Such a dependence is typical for the Brillouin and Rayleigh SLS [37]. The SLS in our case is essentially orientational since the optical nonlinearity mechanism is related to the SALC layer displacement and occurs without the mass density change [15]. For the feasible optical wave electric fields *E* the condition $\varepsilon_{0}E^{2}/B\ll 1$ is always valid, and the gain saturation does not take place.

**Figure 7:**
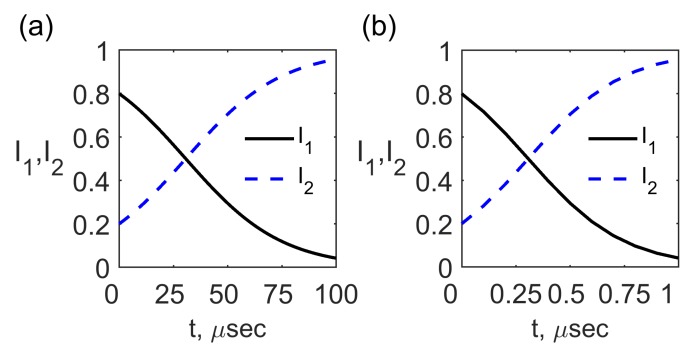
The time dependence of the normalized intensities $I_{1,2}\left(t\right)$ for the initial conditions $I_{1}\left(0\right)=0.8$, $I_{2}\left(0\right)=0.2$, pumping wavelength $\lambda_{1}=1.55$ μm and the pumping wave electric field amplitude $E_{0zSA1}=10^{5}$ V/m (**a**) and $10^{6}$ V/m (**b**).

It is seen from expressions (59) and (60) that for t→∞ the pumping wave intensity is depleted $I_{1}\left(t\right)\to 0$ while the signal wave intensity is amplified up to the saturation level $I_{2}\left(t\right)\to 1$. The time dependence for the normalized intensities $I_{1,2}\left(t\right)$ for the initial conditions $I_{1}\left(0\right)=0.8$, $I_{2}\left(0\right)=0.2$, pumping wavelength $\lambda_{1}=1.55$ μm and the pumping wave electric field amplitude $E_{0zSA1}=10^{5}$ V/m, $10^{6}$ V/m is shown in Figure 7. It is seen from Figure 7a,b that the amplified signal wave rise time is about 60 μsec and 0.6 μsec for the feasible electric field ∼$10^{5}$ V/m, $10^{6}$ V/m, respectively, which is much faster than the director axis relaxation time $\tau_{r}∼1$ ms in NLC [14].

Integrating both parts of Equations (51) and (52) over *z* from −d up to *d* and substituting expressions (58)–(60), (62) into these equations, we obtain the expressions for the pumping and signal wave phases $\theta_{1,2}$. They have the form.

(65) θ1t=ReGk,β,Δω2ImGk,β,ΔωlnexpgFkdt1−I10+I10

(66) θ2t=−ReGk,β,Δω2ImGk,β,ΔωlnI10exp−gFkdt+1−I10

It is seen from Equations (65) and (66) that XPM occurs, and the depletion of the pumping wave is accompanied by rapid linear increase of its phase $\theta_{1}\left(t\right)$ which corresponds to the fast oscillations of the amplitude $E_{0zSA1}$.

(67) t→∞,θ1t≈ReGk,β,Δω2ImGk,β,ΔωgFkdt→∞

The phase of the amplified signal wave $\theta_{2}\left(t\right)$ tends to the constant level:

(68) t→∞,θ2t→−ReGk,β,Δω2ImGk,β,ΔωlnI20

**Figure 8:**
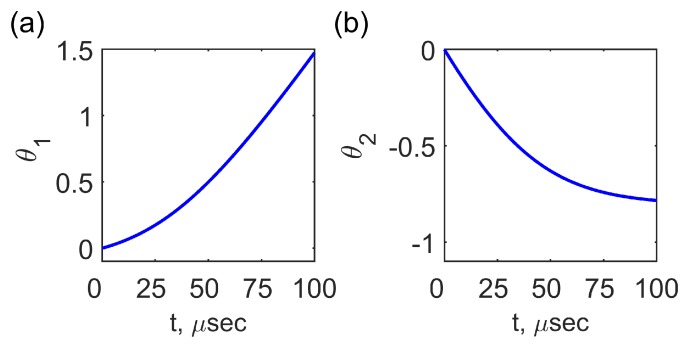
The temporal evolution of the phases $\theta_{1}\left(t\right)$ (**a**) and $\theta_{2}\left(t\right)$ (**b**) for the pumping wave electric field amplitude $E_{0zSA1}=10^{5}$ V/m and the pumping wavelength $\lambda_{1}=1.33$ μm.

The temporal evolution of $\cos\theta_{1,2}\left(t\right)$ is shown in Figure 8a,b, respectively. The characteristic time of the phase variation is about $10^{-4}$ s for the pumping wave electric field amplitude $E_{0zSA1}=10^{5}$ V/m. The comparison of expressions (38), (61), (65) and (66) shows that in the SS resonance case Δω=Ω, ReGk,β,Δω=0, XPM is absent: $\theta_{1,2}=const$.

Consider now the hydrodynamic behavior of the SALC core. Substituting expressions (48), (58) and (64) into expression (37) we obtain the explicit expression of the smectic layer grating amplitude $U_{0}$.

(69) $$U_{0}=\frac{\varepsilon_{0}\beta^{2}kI_{0}\sqrt{\omega_{1}\omega_{2}}\left[\varepsilon_{a}\frac{\varepsilon_{∥}}{\varepsilon_{⊥}}+a_{⊥}\frac{1}{2}{\left(\frac{k\varepsilon_{∥}}{\beta \varepsilon_{⊥}}\right)}^{2}+\frac{1}{2}a_{\|}\right]}{4\rho \left(\beta^{2}+k^{2}\right)G\left(k,\beta ,\Delta \omega \right)}\frac{\exp i\left(\theta_{1}-\theta_{2}\right)}{\cosh\left[\frac{1}{2}gF\left(kd\right)\left(t-t_{0}\right)\right]}$$

It is seen from Equation (69) that the crossing time $t_{0}$ corresponds to the maximum of the smectic layer strain pulse. Substituting expressions (36) and (69) into Equations (6) and (7) we obtain the following expressions of the hydrodynamic velocity components $v_{x,z}\left(x,z,t\right)$.

(70) $$v_{x}=\frac{k\Delta \omega }{\beta }U_{0}\cos2kz\exp i\left[\left(\omega_{1}-\omega_{2}\right)t-2\beta x\right]+c.c.$$

(71) $$v_{z}=i\Delta \omega U_{0}\sin2kz\exp i\left[\left(\omega_{1}-\omega_{2}\right)t-2\beta x\right]+c.c.$$

It is seen from expressions (70) and (71) that they also have the form of the pulses (69).

## 5. Conclusions

We investigated theoretically the nonlinear optical phenomena in the optical slab waveguide with the SALC core. We calculated the TM and TE modes in such a strongly anisotropic waveguide. We have shown that the single mode regime can be realized for the waveguide core thickness of about 1–2 μm and optical wavelength of $\lambda_{opt}∼1.35$–1.55 μm important for the optical communication applications. The cubic nonlinearity of SALC is related to the smectic layer normal displacement. The nonlinear interaction is especially strong for the counter-propagating TM modes. We solved simultaneously the equation of motion for the smectic layer normal displacement in the optical field and the wave equation for the TM mode electric field using SVA approximation. The interfering optical fields create the smectic layer displacement dynamic grating which propagates in SALC without the mass density change. As a result the nonlinear polarization occurs and the SLS accompanied by XPM takes place in the waveguide. We evaluated the pumping and signal TM mode SVA magnitudes and phases. In the resonance case when the TM mode frequency difference Δω equals to the SS frequency Ω the gain *g* has a maximum value, and XPM is absent. The smectic layer strain has a pulse form with a maximum corresponding to the crossing time of the pumping and signal TM modes. We also evaluated the hydrodynamic velocity enhanced by the interfering TM modes. The numerical estimations show that the SLS in SALC is much faster than the light scattering in NLC related to the director reorientation.
